# Facts about the history of the AE&M

**DOI:** 10.1590/2359-3997000000297

**Published:** 2017-07-01

**Authors:** Antonio R. Chacra

**Affiliations:** 1 Centro de Pesquisa Clínica em Diabetes Escola Paulista de Medicina Universidade Federal de São Paulo São Paulo SP Brasil Diretor do Centro de Pesquisa Clínica em Diabetes da Escola Paulista de Medicina da Universidade Federal de São Paulo (EPM/Unifesp). Diretor do Centro de Diabetes do Hospital Sírio- -Libanês, São Paulo, SP, Brasil

The *Archives of Endocrinology and Metabolism –* AE&M (formely ABE&M – *Arquivos Brasileiros de Endocrinologia e Metabologia*) is the official scientific journal of the Brazilian Society of Endocrinology and Metabolism (Sociedade Brasileira de Endocrinologia e Metabologia – SBEM) which is affiliated with the Brazilian Medical Association (Associação Médica Brasileira).

Besides SBEM, the alma mater society, the journal also provides a connection for publications with the following medical organizations and associations of interest in the fields of Endocrinology and Metabolism: Brazilian Diabetes Society (Sociedade Brasileira de Diabetes – SBD) and the Brazilian Association for the Study of Obesity (Associação Brasileira para o Estudo da Obesidade – ABESO).

This journal is published on a regular basis, with six printed issues per year in addition to online version at AE&M homepage and SciELO. Special issues on seleted topics as well the proceedings of the Brazilian Endocrinology Congresses are also published. It is a peer reviewed journal with a very scientifically respected Editorial Board. AE&M (ISSN 2359-3997 – printed, ISSN 2359-4292 – on line) is indexed on PubMed^®^/MEDLINE^®^ U.S. National Library of Medicine, ISI-Web of Science, SCOPUS, BIREME-LILACS, Excerpta Medica, Chemical Abstracts, Biological Abstracts, Academic Google.

This year we celebrate the 66^th^ anniversary of the foundation of the AE&M. It was with a great joy and surprise that I received a phone call from Marcello Bronstein, the current Editor-in-Chief of the AE&M, asking to write an editorial commenting on some historical facts about the AE&M. I immediately agreed because I do believe that the knowledge of how the Archives have reached this high level of a great scientific journal have developped over the years is important for the continuing progress of the journal.

We do not know exactly who conceived the idea of launching a Brazilian journal in the field of endocrine glands. Around the nineteen fifties, there was a pletora of basic scientists in the axis Rio de Janeiro-São Paulo working in the area of internal secretion. Eventually the ABE&M was founded in 1951 by this basic scientist group together with another group of distinguished clinical medical doctors. These scientists doctors are considered the pioneers of Endocrinology in Brazil. They were either envolved in basic science or in clinical endocrinology. In the first group, we should mention Thales Martins and Ribeiro do Valle. Endocrinology at that time was commencing to be characterized as a clinical specialty. Among these medical doctors interested in endocrine disease we should cite Emilio Mattar, João Gabriel H. Cordeiro, Luciano Décourt, Arnaldo Sandoval, Luiz Carlos Lobo and many others, specially from Rio de Janeiro. Regarding the foundation of the ABE&M, all of them were represented by Waldemar Berardinelli, a famous endocrinologist from Rio, who is considered the founder and was the first Editor-in-Chief of the journal. Interestigly, the foundation year (1951) of the Archives was also the year that the *Journal of Clinical Endocrinology and Metabolism* (JCEM) was founded by the Endocrine Society in the United States. Therefore the ABE&M together with the JCEM is one of the oldest medical journal in the field of Endocrinology.

We do not know exactly what came first, the society (SBEM) or the journal (ABE&M). But certainly, that time was the heyday of the growth of Endocrinology and the turning point for the recognition of Endocrinology as a medical specialty.

Founder, Previous Editors and presente Editor

Waldemar Berardinelli (Founder Editor) 1951-1955

Thales Martins, 1951-1955

Clementino Fraga Filho, 1957-1963

Luiz Carlos Lobo, 1964-1966

Pedro Colletti-Solberg, 1966-1968

João Gabriel H. Cordeiro, 1969-1972

Armando de Aguiar Pupo, 1978-1982

Antonio Roberto Chacra, 1983-1990

Rui Monteiro de Barros Maciel, 1991-1994

Claudio E. Kater, 1995-2006

Edna T. Kimura, 2007-2010

Sergio Atala Dib, 2011-2014

Marcello D. Bronstein, 2015-present

We can divide the history of the AE&M in diferente stages accordingly to the tenures of the Editors-in-Chief. The first years are charecterized for publishing mainly the results of original articles related to experimental research. As the clinical specialty of Endocrinology emerged, many clinical articles in the field of physiopathology, diagnosis and treatment of endocrine and metabolic diseases started to increase. For reasons not very well clarified, there was a gap of publications from 1973 to 1977.

In 1978, Armando de Aguiar Pupo was indicated as Editor-in-Chief. I remember the great effforts of Armando Pupo to revive the journal, proactively asking us to send manuscripts to be submitted and then published at the Archives. Armando Pupo can be considered the co-founder of this new phase of the ABE&M. We have to be very grateful to Armando Pupo for his dedication in revival the Archives

I followed Pupo as the Editor-in-Chief (1983-1990). During those years Brazil was in economic turmoil and recession, with a high rate of inflation. My main objective at that time was raising funds in order to save the Archives and keep the publication of the journal. I wrote a project and I remember sending the Archives project to a great number of organizations, including pharmaceutical companies. Finances were very bad at that time and we received a lot of refusals. Eventually we got a support from the Brazilian Medical Association and the CNPq (*Conselho Nacional de Pesquisa*). I am very grateful to CNPq, specially in the person of Reginaldo Holanda e Albuquerque, a great endocrinogist from Brasilia, who helped us to keep the journal alive. During that time, I had also to deal with the legal formalities of the journal and the connection of the Archives to our Brazilian Society of Endocrinology and Metabolism (SBEM). Another task was to make the Archives known to new subscribers and contributors and stimulate receiving manuscripts for publication.

Rui Monteiro de Barros Maciel followed me being indicated in 1990 as the new Editor-in-Chief. I remember how glad he was by this nomination. Full of enthusiasm, Rui Maciel had, as one of his main objectives, to use the Archives as a tool to ameliorate the professional capacity of the endocrinologists in Brazil. He stimulated the publications of clinical reviews, cases reports, clinical articles as well clinical guidelines. He was also responsible for introducing editorials commenting the data of original articles. We have also to mention that it was during his tenure that special issues in selected topics started to be published, as well the proceedings of the Brazilian Congresses of Endocrinology and others scientific events organized by SBEM. At that time the Archives also started to receive a great number of manuscripts related to the Post Graduate Courses that have been implanted in Brazil. He acted in a very proactive manner asking colleagues to submitt papers to the Archives.

In a personal interview, Rui Maciel pointed out how proud he is for had served as Editor-in-Chief of the Archives, and that he also considers this position as one of the crucial point on his career.

After Rui M. B. Maciel the next Editor-in-Chief was Claudio E. Kater. One of the higlights of this period was the starting process of indexation of the journal. This was a very hard work that without Kater would not be possible.

The period of Edna T. Kimura (2007-2010), who followed Kater, was one of the most proficuous in the history of the Archives. In 2009, Edna Kimura expanded the fascicles number/year from six to nine, a great achiement.

The following remarkable facts were also achieved during her period and deserve to be mentioned: eletronic edition on the site, www.abem-sbem.org.br was made publically available extending the visibility of ABE&M, that was electronically visible also in the www.scielo.br. Eletronic Submission in 2008-January. First JCR-Web of Science Impact Factor in 2009. Financial support of CNPq (2007-2010). Edna also gave continuity to the publication of the tematic esdtions.

Sergio Atala Dib was the next Editor-in-Chief. Regarding the rules for publication, Sergio Atala Dib during his tenure introduced an important issue against plagiarism. That was a great achievement among others that Dib has contributed for the Archives. After completing his four year period as Editor-in-Chief, he wrote a editorial where he enphasised the importance of this measure: “In order to regulate the publishing procedures in ABE&M after publication, the publishing authorization and the copyright cession term, a tool for the transference of the copyright to ABE&M, was created with the aid of the Juridical Department of the Brazilian Society of Endocrinology and Metabolism. In this era when publications became profuse and sophisticated, there is an increase in the prevalence of plagiarism, including in journals of high impact factor. In order to protect ABE&M from plagiarism, an anti-plagiarism tool was recently acquired, and all manuscripts will be analyzed by it before being published”.

The more recent and innovative phase on the history of the Archives started by the time Marcello D. Bronstein was apointed as Editor-in-Chief at the beggining of 2015. Bronstein totally transformed the journal into an international one. The first step was changing the name to “*Archives of Endocrinology and Metabolism*” (AE&M). As he wrote in his first editorial, “the aim was to make Brazil even more visible in the international scientific community. In fact, I expect that, little by little, colleagues in Brazil and abroad give prestige to our journal by submitting high level articles. This will only be possible if AE&M, by raising its impact fator”. The result of that change has been positive and a great number of articles from several countries around the world have been published. Brazilian authors, as well, are now submitting more and more articles to AE&M.

Finally, I would like to congratulate Marcello D. Bronstein for his great achievement as the present Editor-in-Chief of the AE&M and to thank him for inviting and giving me the opportunity of writing this editorial.

